# A protocol of hepatic volume measurement using magnetic resonance imaging in individuals from the Eastern Brazilian Amazon population

**DOI:** 10.1371/journal.pone.0229525

**Published:** 2020-03-05

**Authors:** Robson Tadachi Moraes de Oliveira, Apio Ricardo Nazareth Dias, Waldônio Brito Vieira, Aline Semblano Carreira Falcão, Luiz Fábio Magno Falcão, Juarez Antônio Simões Quaresma

**Affiliations:** 1 Fundação Santa Casa de Misericórdia, Belém, PA, Brazil; 2 Núcleo de Medicina Tropical, Universidade Federal do Pará, Belém, PA, Brazil; 3 Centro de Ciências Biológicas e da Saúde, Universidade do Estado do Pará, Belém, PA, Brazil; 4 Instituto Evandro Chagas, Ananindeua, PA, Brazil; University of Tsukuba, JAPAN

## Abstract

Determination of hepatic volume is an important preoperative procedure and is done through imaging exams or standard liver volume (SLV) formulas developed based on the biotype of each population. In the absence of a specific SLV formula for the Brazilian Eastern Amazon population, the measurement of liver volume is made with reference values from other populations. The aim of study was to compare the hepatic volume in healthy residents from the Brazilian Eastern Amazon population obtained with magnetic resonance imaging (MRI) and recommended SLV formulas validated to other populations. This was a Observational, cross-sectional study. Anthropometric data of 42 healthy individuals aged 18–60 years of both sexes was collected to measure the liver volume through SLV formulas calculations and MRI. MRI shows similarity with the Western European SLV liver volume values and significant differences with the Japan SLV formula, mainly for women, with a moderate-to-weak correlation with the MRI measurements. There was a strong correlation between weight and body surface area in male patients analysed with measurements of the liver volume by the MRI and SLV formulas. The SLV formula based on the Western European population could be used in the absence of a specific formula for individuals living in the Amazon region. The results suggest that liver measurement formulas should take into consideration the sex of individuals, as well as the development of a specific SLV formula for the Eastern Amazon population and the conduction of similar studies in other Brazilian regions.

## Introduction

In the Brazilian Eastern Amazon Region, the prevalence of chronic viral hepatopathies in the general population ranges from 1.1 to 2.4% and within blood donors it ranges from 0.8% to 5.9%, the State of Pará having one of the higher rates in the Region with 2% affected individuals [1]. Due to the natural evolution to cirrhosis of chronic viral hepatopathies, imaging methods such as ultrasonography, tomography, and magnetic resonance are frequently employed to define hepatic dimensions, but the diagnosis of hepatomegaly or a reduced liver volume are commonly based only on the measurements of the right and left lobes, which do not always define the liver volume satisfactorily [2].

In procedures such as segmentectomies and liver transplantations, the amount of donated and remaining liver tissues has a direct implication on the prognosis of those involved; thus, the precision of hepatic dimension measurements conducted through computed tomography or MRI, which is more reliable, is very important [3,4].

The definitions of normal hepatic dimensions are dependent on the population studied. Brazil is characterized by miscegenation between Europeans, blacks and indigenous people. There are fluid sociability relationships with little racial segregation between whites and blacks, and large numbers of interracial marriages [5]. According to the last Brazilian census, Pará is the state of the federation with the largest number of people who call themselves brown or black [6].

Hepatic volumetry is a method with greater sensitivity and specificity. Usually hepatic volumetry is calculated by computed tomography (CT). However, other modalities for this measurement, such as ultrasonography (USG) and magnetic resonance imaging (MRI), have been described, being more safety modalities than CT, a radiologic method [7].

The precise determination of the hepatic volume, especially in cirrhotic patients (where it is a prognostic factor), in the selection of living transplants donors (where it has a key role), and in the preoperative planning of primary tumours or metastases resections is imperative.[8] Hepatic volumetry has also been applied in the determination of the postoperative residual hepatic volume, where it is related to the chance of a future development of hepatic impairment and mortality [9].

The absence of data regarding hepatic volume determination in individuals living in the Brazilian Eastern Amazon region brings uncertainty to the preoperative evaluation in inter-vivos transplant programs of the State of Pará, since the formulas used for this calculation are related to the biotype of other populations.

In this study were investigated the mean hepatic volume values of healthy individuals from Brazilian Eastern Amazon population correlating the mean values calculated by MRI with the mean values calculated through the Standard Liver Volume (SLV) formula for the Japanese population (SLV Japan) and for the Western Europe populations (SLV Europe).

## Materials and methods

### Study design and population

This was a cross-sectional observational study conducted with healthy individuals of both sexes, aged between 18 and 60 years old and born, and residing in the state of Pará.

### Sample calculation

The sample size to compare the different acquisition methods of liver volumetry for Eastern Amazon Population was calculated after a pilot study with twelve patients. The t-test was applied for two dependent samples, considering the means (1403.9 ± 95.8 cm^3^, 1252.3 ± 102.3 cm^3^, and 1414.7 ± 183.6 cm^3^) of the hepatic volume measurements obtained by MRI, SLV formula for the Japanese population, and SLV formula for the European population, respectively. The power of the test was of 90%, with a confidence interval of 95% and the alpha level of 0.05, obtaining an initial estimated sample of 42 individuals. Bioestat 5.0 ^TM^ (Sociedade Civil Mamirauá, Manaus, Brazil) was the statistical software utilized.

### Inclusion criteria

The study included healthy patients who signed the Informed Consent Form, with liver anatomically positioned and without structural deformities defined by the MRI of the upper abdomen, aged between 18 and 60 years, and residents of the State of Pará.

### Exclusion criteria

Patients with hepatopathies such as viral, toxic, drug, acute bacterial or parasitic hepatitis, those with signs of focal and / or diffuse hepatopathy proven by examinations of the upper abdomen before or during study, with liver deformities and those who refused to sign the Informed Consent Form were excluded from the study.

### Study location

The research was carried out in the Medical Radiology Service of the “Fundação Santa Casa de Misericórdia do Pará (FSCMPA)”, which is a hospital linked to the State Department of Public Health.

### Data gathering

The volunteers were recruited and after signing the informed consent form they responded to a questionnaire referring to clinical signs and symptoms, presence of associated diseases, previous laboratory exams, and medications in use. The weight, height, biotype, and body mass index (BMI) of the individuals participating in the study were determined. Weight and height were measured on a calibrated scale, while the BMI was calculated using the following formula: BMI = weight (kg)/height^2^ (m^2^). The standard liver volume was estimated by the medical specialist from the service using the formulas based on the Japanese (SLV = 2.4 + 706.2 × BSA) and Western Europe populations (SLV = -794.41 + 1267.28 × BSA) [10,11]. Body surface area (BSA) was obtained using the formula: BSA (m^2^) = 0.007184 × (Height (cm) ^0.725^) x (Weight (kg) ^0.425^), prior to performing MRI.

After that, each participant underwent an MRI using equipment of 1.5 Tesla (GE Healthcare, Chalfont St Giles, UK) previous calibrated. To obtain transverse images of the abdomen, the procedure was performed with the individual in the supine position, patients were instructed to remain static and each participant completed a single test without the administration of drugs or chemicals such as sedatives and / or intravenous contrast substance.

**Fig 1:**
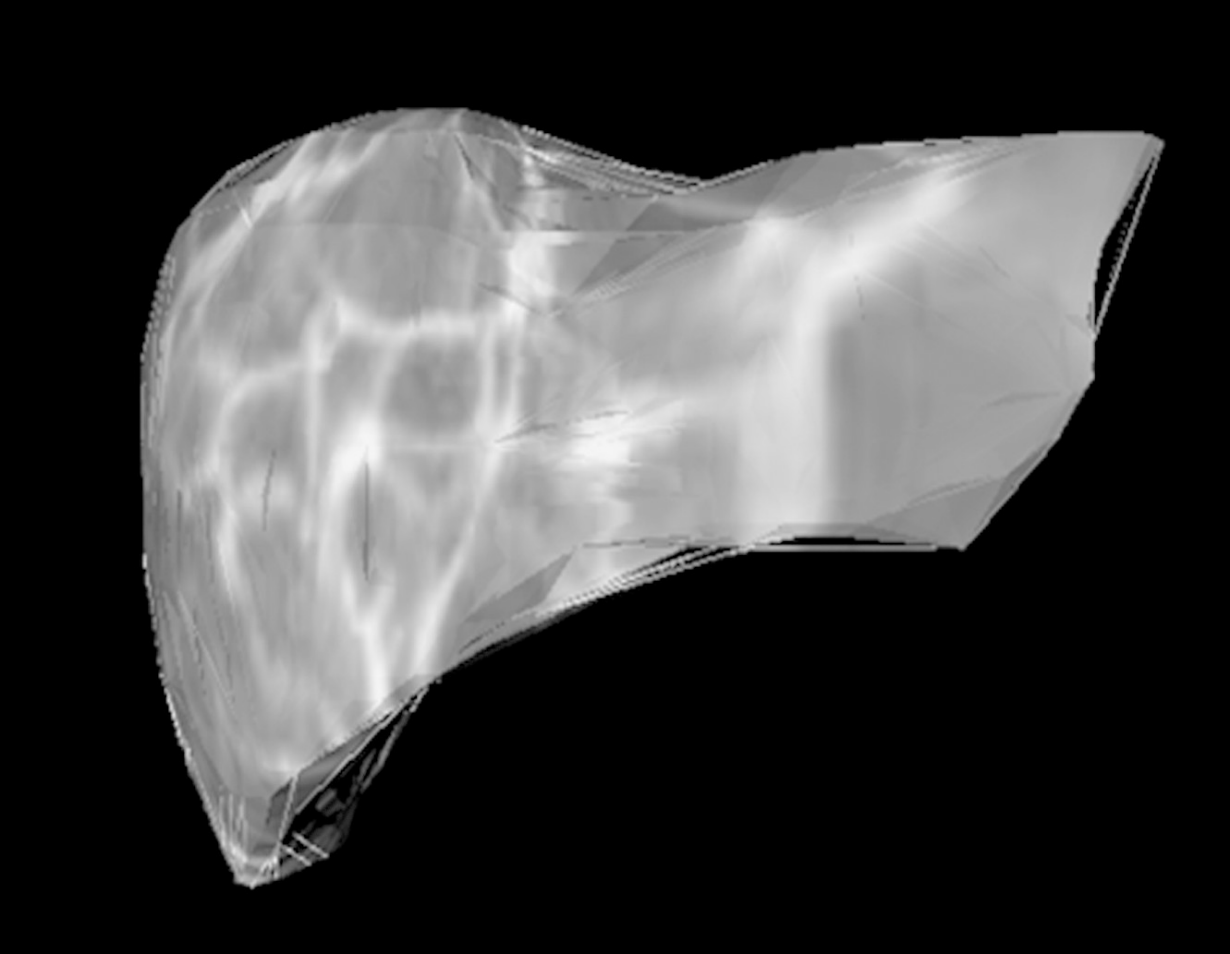
3D image acquired by magnetic resonance imaging.

A medical specialist from the service was trained to made the procedure of hepatic volume measurement using the image analysis software Osirix MD version 5.8.5^TM^ (Pixmeo Company, Bernex, Switzerland) and a computer model Imac^TM^ (Apple Inc., Cupertino, USA) according to the following procedure: LAVA axial sequence was selected and, through the Pencil tool, the peripheral margin of the whole liver was demarcated with the mouse in all MRI cuts, except for the vascular areas. Then, the Region of Interest (ROI) command was selected in the toolbar, ROI compute volume (volume of interest area), which automatically performed the calculation of the hepatic volume, generating a three-dimensional (3D) image (Fig 1).

### Statistical analysis

The collected data was stored in Excel 2007^TM^ spreadsheets (Microsoft Corporation, Redmond, USA) and analysed using the BioStat 5.0^TM^ (Sociedade Civil Mamirauá, Manaus, Brazil) software and GraphPad Prism 5.0 (GraphPad Software, San Diego, USA), including the graphic design.

To compare the measured values with those predicted by the equations proposed to Japanese population [10] and Western Europe population [11] the Shapiro-Wilk test was applied to evaluate the normal distribution. The Student t-test was used for variables with a normal distribution, whereas the Wilcoxon test was adopted for the variables that did not present a normal distribution. A significance level of 5% (α < 0.05) was adopted for rejection of the null hypothesis.

Correlations between hepatic volumes and between the independent variables (weight, height, and BMI) were determined using the Pearson linear correlation coefficient and displayed by means of a scatter diagram.

### Ethical aspects

The research was submitted to analysis by the Research Ethics Committee of the FSCMPA and approved under protocol number 1074368. In the development of the research, the Nuremberg Code and Declaration of Helsinki precepts were respected, as well as the Research Norms Involving Human Beings (Res. CNS 466/12) and its complementary National Health Council (CNS) / Brazilian Ministry of Health. All the participants signed an Informed consent form, declaring that they were aware of the research procedures and authorizing the collection of data.

## Results

The hepatic volume values were obtained using the MRI method and the SLV formula for Western European and for Japanese populations (Table 1).

**Table 1:** Characteristics of healthy individuals volunteers.

Volunteer	Sex/Age	Wt (kg) /Ht (cm)	BMI	MRI (cm^3^)	SLV Japan (cm^3^)	SLV Europe (cm^3^)	RHL (cm)	LHL (cm)	BSA (m^2^)
#1	F/28	57.0/175	18.61	1013.77	1198.43	1351.88	12.230	10.248	1.6936
#2	F/45	60.0/162	22.86	1014.40	1158.26	1279.79	13.761	7.371	1.6367
#3	F/55	52.0/155	33.54	1047.58	1055.78	1095.89	14.024	7.481	1.4916
#4	F/33	50.6/154	21.33	1050.69	1038.76	1065.34	13.332	7.299	1.4675
#5	F/30	64.0/191	28.06	1086.73	1341.06	1607.82	13.124	6.397	1.8956
#6	F/47	63.0/168	22.32	1096.96	1214.01	1379.83	14.014	8.720	1.7157
#7	F/56	56.0/150	24.88	1104.56	1063.95	1110.55	14.774	7.779	1.5032
#8	F/37	63.0/155	26.22	1105.23	1145.29	1256.51	12.851	6.081	1.6184
#9	F/35	60.0/155	24.97	1141.25	1121.83	1214.42	14.247	7.245	1.5852
#10	F/27	64.0/158	25.63	1141.90	1169.07	1299.18	14.677	6.596	1.6520
#11	F/23	58.0/153	21.82	1170.69	1095.48	1167.13	13.241	8.175	1.5478
#12	F/39	63.0/160	24.60	1187.10	1171.90	1304.27	15.536	7.448	1.6560
#13	F/27	72.0/168	25.51	1245.10	1284.76	1506.79	14.851	6.432	1.8159
#14	M/35	73.0/172	24.67	1257.37	1314.49	1560.15	14.332	9.104	1.8580
#15	F/21	80.8/163	30.11	1262.01	1319.99	1570.01	13.788	7.267	1.8657
#16	F/23	61.0/162	23.24	1273.21	1166.41	1294.42	13.740	6.129	1.6483
#17	F/33	79.0/158	31.64	1302.55	1278.29	1495.18	15.789	7.416	1.8067
#18	M/50	67.0/168	23.73	1314.94	1246.13	1437.47	15.752	8.561	1.7612
#19	M/24	73.0/175	23.83	1318.47	1331.04	1589.85	15.456	8.026	1.8814
#20	F/43	58.0/165	21.67	1319.63	1156.99	1277.50	14.979	9.354	1.6349
#21	F/47	66.0/168	23.38	1341.48	1238.20	1423.25	15.300	7.128	1.7499
#22	M/50	77.0/180	23.76	1364.01	1389.56	1694.85	16.303	7,498	1.9643
#23	F/38	55.0/150	24.44	1364.12	1055.85	1096.01	14.173	8.230	1.4917
#24	F/53	75.0/156	30.81	1416.79	1238.95	1424.58	16.217	7.035	1.7510
#25	F/33	62.0/169	21.70	1509.36	1210.99	1374.41	14.597	7.849	1.7114
#26	M/25	88.0/176	28.40	1514.47	1446.83	1797.62	16.592	7.600	2.0454
#27	F/41	72.0/158	28.84	1526.17	1228.95	1406.65	15.596	8.340	1.7368
#28	F/54	65.0/162	24.76	1555.30	1198.26	1351.57	14.670	8.168	1.6934
#29	M/40	69.8/167	25.02	1556.03	1262.49	1466.83	15.645	8.304	1.7843
#30	M/51	66.0/163	24.84	1565.95	1211.43	1375.20	16.029	7.115	1.7120
#31	F/31	75.0/170	25.95	1586.73	1318.44	1567.24	14.810	10.909	1.8636
#32	F/31	68.0/165	24.97	1615.92	1237.74	1422.41	15.239	11.898	1.7493
#33	M/33	75.0/174	24.77	1616.88	1340.82	1607.40	17.380	9.596	1.8952
#34	M/39	88.0/177	28.08	1624.48	1452.77	1808.29	16.751	7.960	2.0538
#35	F/34	77.0/169	26.95	1632.51	1327.57	1583.61	16.256	8.330	1.8765
#36	F/44	76.0/160	47.50	1666.27	1268.96	1478.44	15.692	7.980	1.7935
#37	M/37	76.0/165	27.91	1672.72	1297.54	1529.72	16.023	10.585	1.8340
#38	M/40	94.0/180	27.17	1754.98	1512.29	1915.10	14.495	7.666	2.1381
#39	M/42	90.0/177	28.72	1843.32	1466.69	1833.27	15.066	8.287	2.0735
#40	F/43	70.0/155	29.13	1995.11	1197.63	1350.44	15.521	11.159	1.6925
#41	M/25	128.0/195	34.72	2300.24	1806.46	2442.99	16.795	10.351	2.5546
#42	M/42	101.0/183	30.15	2368.40	1577.86	2032.76	18.911	10.160	2.2309

Wt: body weight. Ht: body height. BMI: body mass index. MRI: hepatic volume measured by Magnetic Resonance Imaging. SLV Japan: hepatic volume measured by Standard Liver Volume formula to Japanese population. SLV Europe: hepatic volume measured by Standard Liver Volume formula to Western Europe population. RHL: right hepatic lobe volume. LHL: left hepatic lobe volume. BSA: body surface area.

### The means of values obtained by the different methods and considering the gender of volunteers were obtained (Table 2)

**Table 2:** Liver volume obtained by different methods.

Method	Both sexes (n = 42)	Male(n = 14)	Females(n = 28)
MRI	1424.8 ± 318.1 cm^3^	1648 ± 337.6 cm^3^	1313.3 ± 245.1 cm^3^
Western Europe SLV	1472.5 ± 269.6 cm^3^	1720.8 ± 282.8 cm^3^	1348.3 ± 154.6 cm^3^
Japan SLV	1265.6 ± 150.2 cm^3^	1404 ± 157.6 cm^3^	1196.4 ± 86.1 cm^3^

MRI: Magnetic resonance imaging. SLV: Standard Liver volume.

**Fig 2:**
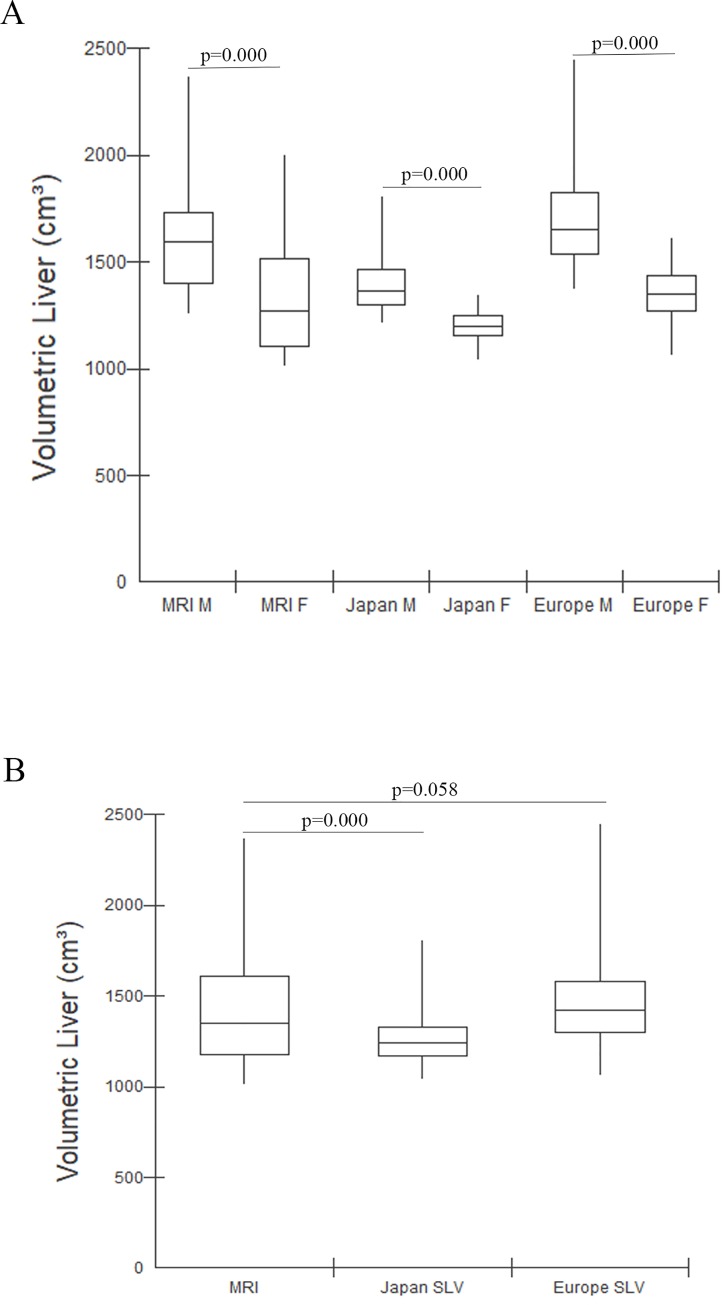
Comparison between mean values of liver volume. (A)Comparison between mean values of Liver volume in male and female healthy individuals, using MRI, Japanese SLV formula and Western Europe SLV formula. (B) Mean value of Liver volume using MRI (μ = 1424.8±318.1 cm^3^), Japanese SLV formula (1265.6 ± 150.2 cm^3^) and Western Europe SLV formula (μ = 1472.5 ± 269.6). MRI: Magnetic resonance imaging, SLV: Standard liver volume.

When considering the hepatic volume measurements by sex, we notice a volume variation according to gender in the study individuals, with smaller hepatic volumes in women. When comparing the mean values of hepatic volume among healthy male and female individuals it is possible to note that all three methods of hepatic measurements showed a significant difference in hepatic volume between sexes (Fig 2).

When comparing the measurements of hepatic volume, independent of sex, using MRI with SLV formulas for the Japanese and Western European populations it is possible to notice that the mean values between the MRI method and the SLV Europe formula have similarity when compared to the SLV Japan formula (Fig 2).

**Fig 3:**
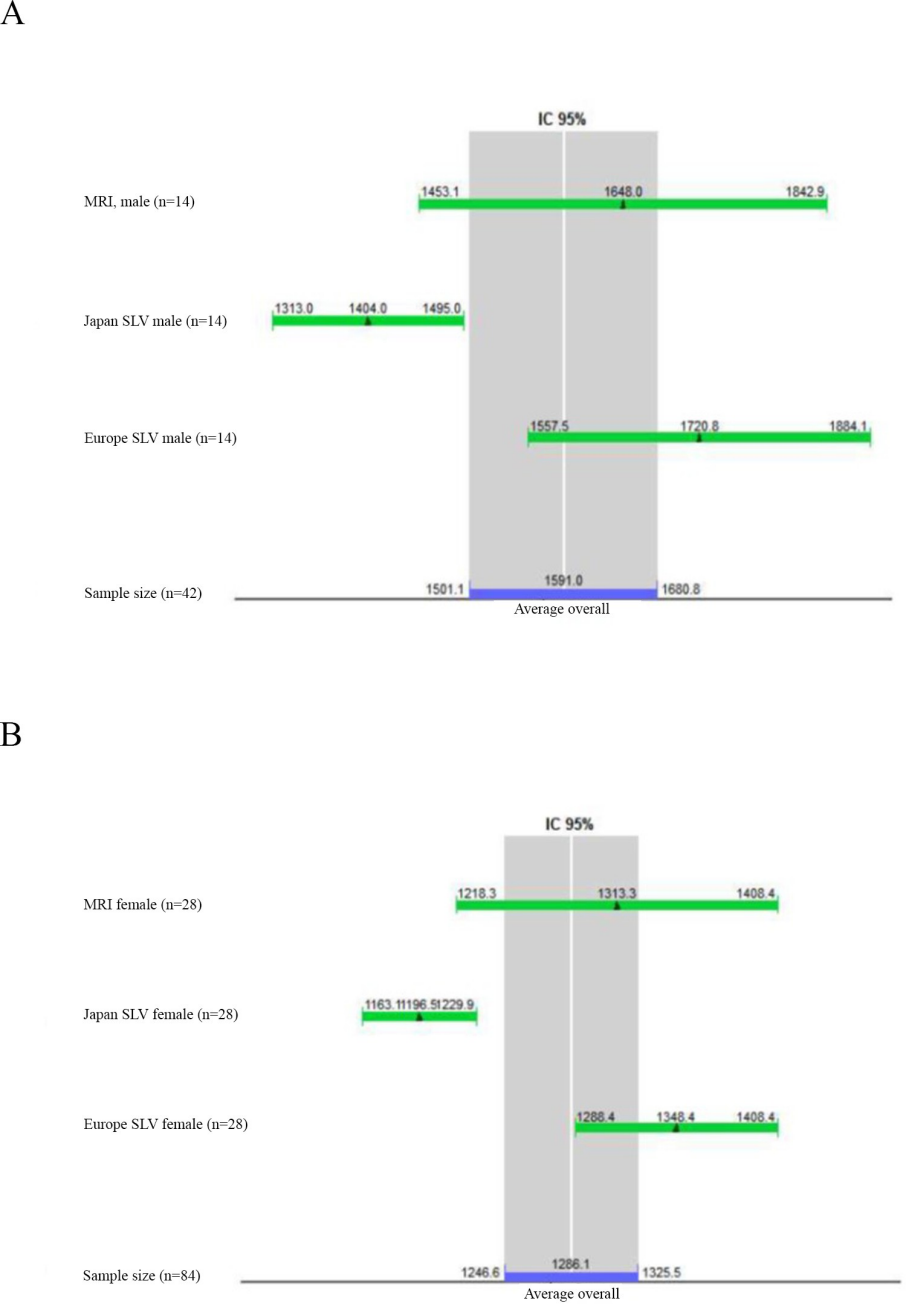
Comparison between mean values and confidence interval of different methods of Liver volume calculation by sex. (A) Comparison between mean values of hepatic volume in male healthy individuals obtained by MRI, Japanese SLV formula and Western Europe SLV formula throught a confidence interval diagram. (B) Comparison between mean values of hepatic volume in female healthy individuals obtained by MRI, Japanese SLV formula and Western Europe SLV formula throught a confidence interval diagram. MRI: Magnetic resonance imaging, SLV: Standard liver volume.

The mean values of hepatic volumetry in healthy male individuals using MRI and SLV Japanese and Western European formulas show that the SLV Japan formula is the most distinguishable from the hepatic measurement performed through MRI, as well as the mean of the general hepatic volume, considering the three methods (Fig 3).

The mean values of hepatic volumetry in healthy female subjects using MRI and SLV formulas applied in the Japanese population and in the Western European population further shows a discrepancy between the proposed values of hepatic volumetry by the Japanese SLV formula in the study population (Fig 3).

**Fig 4:**
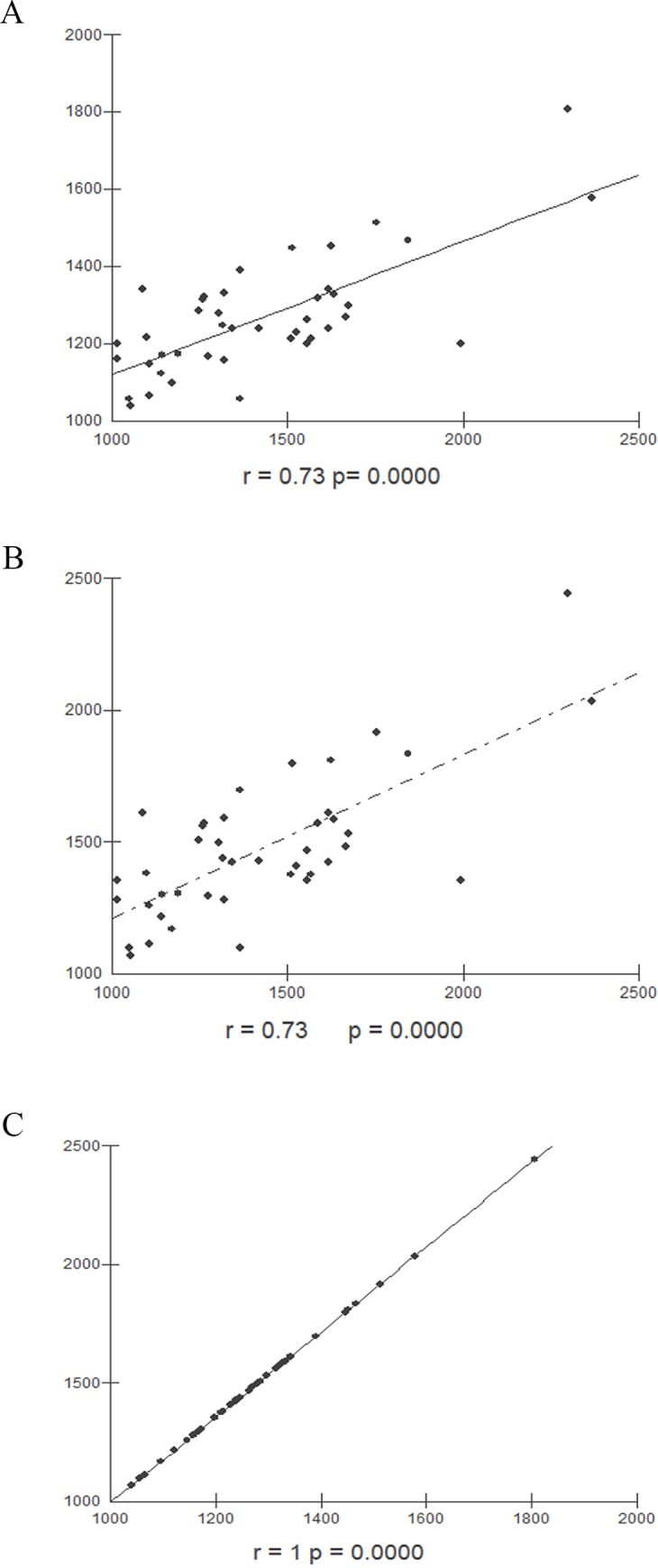
Correlation between hepatic volumes obtained by different methods. (A) Correlation between hepatic volume values obtained by MRI and Japanese SLV formula (r = 0.73, p<0.0001). (B) Correlation between hepatic volume values obtained by MRI and SLV formula for Western Europe population (r = 0.73, p<0.0001). (C) Correlation between hepatic volume values obtained by SLV formulas applied to japanese population and Western Europe population (r = 1, p< 0.0001). MRI: Magnetic resonance imaging, SLV: Standard liver volume.

The values of hepatic volumetry measured by MRI and both SLV formulas Japanese and Western European presented a strong correlation, and the values of hepatic volume measured using the SLV formulas applied for the Japanese and Western European populations was perfect (Fig 4).

**Fig 5:**
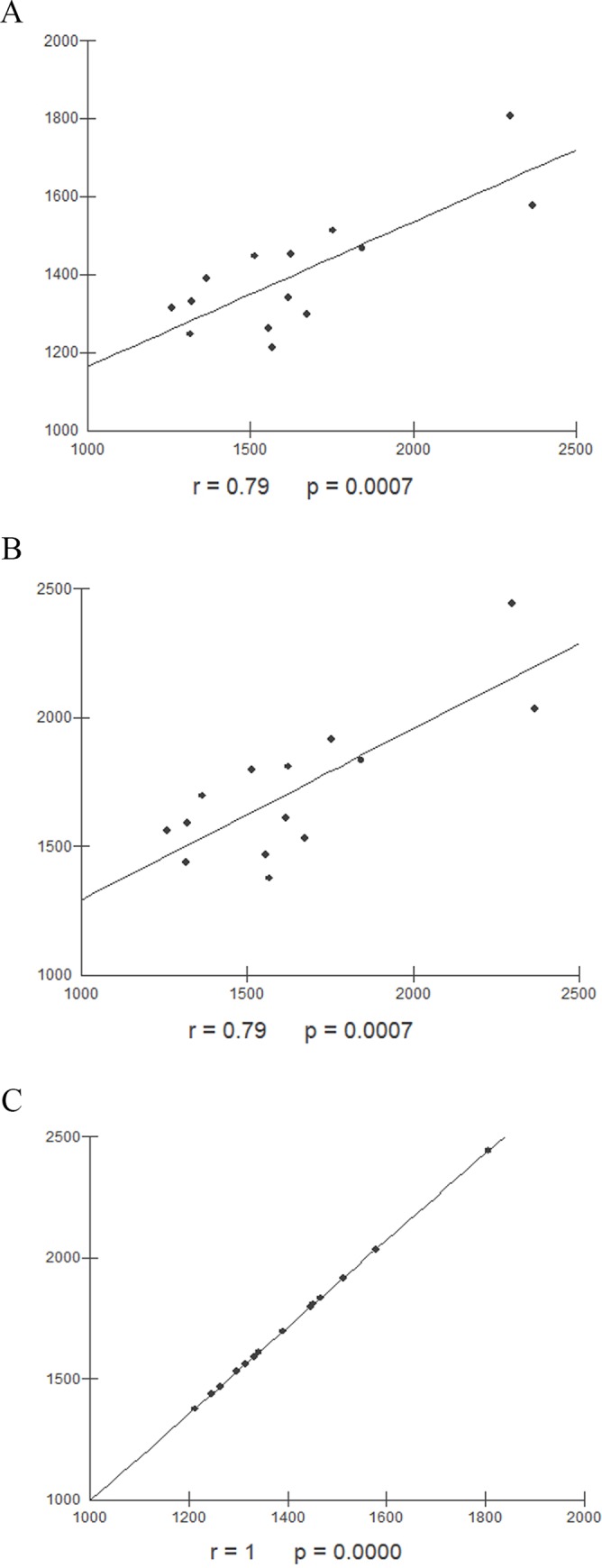
Correlation between hepatic volumes obtained by different methods in male individuals. (A) Correlation between hepatic volume values in male healthy individuals obtained by MRI and Japanese SLV formula (r = 0.79, p = 0.0007). (B) Correlation between hepatic volume values in male healthy individuals obtained by MRI and SLV formula for Western Europe population (r = 0.79, p = 0.0007),. (C) Correlation between hepatic volume values in male healthy individuals obtained by SLV formulas applied to japanese population and Western Europe population (r = 1, p<0.0001). MRI: Magnetic resonance imaging, SLV: Standard liver volume.

In healthy male individuals, the hepatic volume measured by MRI and both SLV formulas, Japanese and Western European showed a strong correlation and a perfect correlation between the SLV formulas Japanese and Western European (Fig 5).

**Fig 6:**
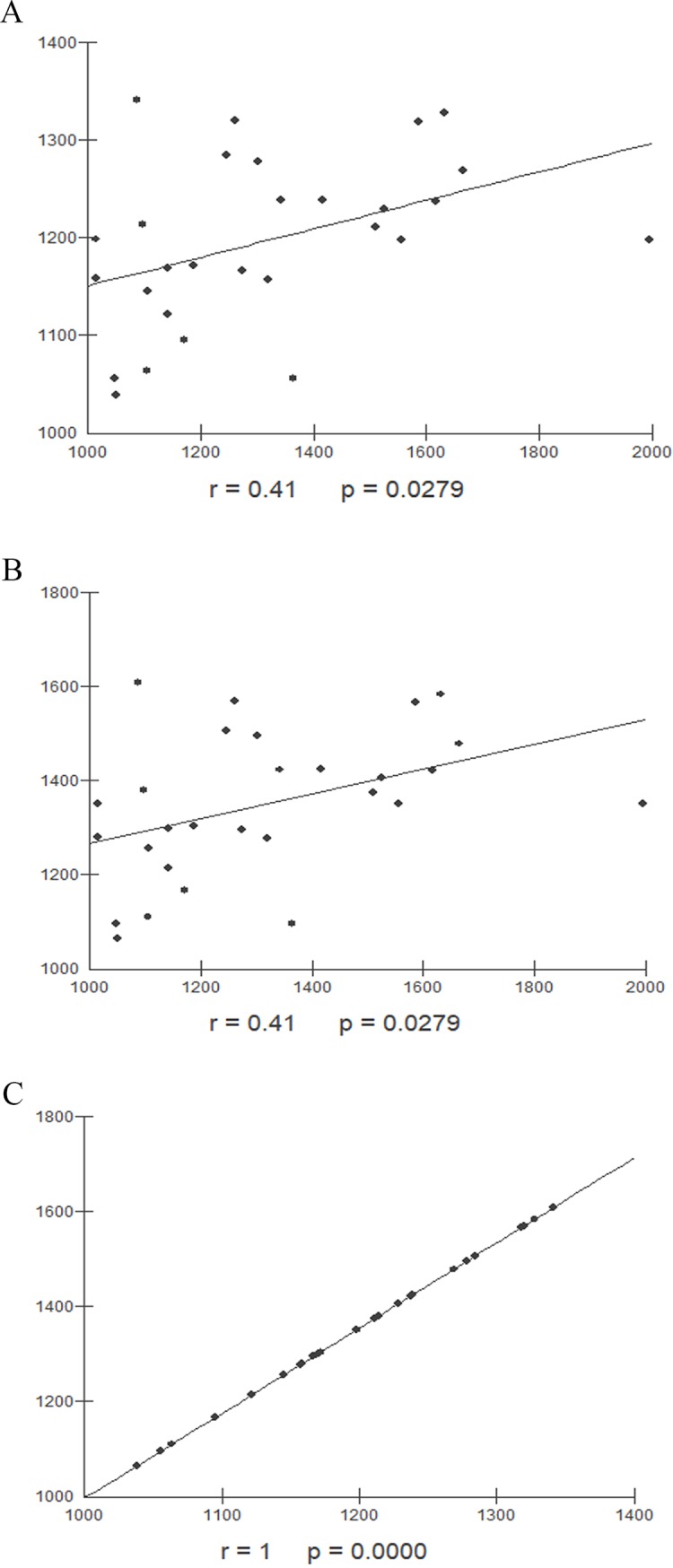
Correlation between hepatic volumes obtained by different methods in female individuals. (A) Correlation between hepatic volume values in female healthy individuals obtained by MRI and Japanese SLV formula (r = 0.41, p = 0.02). (B) Correlation between hepatic volume values in female healthy individuals obtained by MRI and SLV formula for Western Europe population (r = 0.41, p = 0.02). (C) Correlation between hepatic volume values in female healthy individuals obtained by SLV formulas applied to japanese population and Western Europe population (r = 1, p<0.0001). MRI: Magnetic resonance imaging, SLV: Standard liver volume.

In females, the correlation between hepatic volume measured by MRI and SLV formulas exhibited a weak correlation, but the comparison between SLV formulas applied in the Japanese and Western populations presented a perfect correlation (Fig 6).

A correlation of hepatic volume values with anthropometric data of healthy individuals was performed (Table 3).

**Table 3:** Correlation of hepatic volume values with anthropometric data of healthy individuals.

Correlation data	Both sexes (n = 42)		Male (n = 14)		Female (n = 28)	
	r (Pearson)	p-value	r (Pearson)	p-value	r (Pearson)	p-value
MRI and Weight	0.79	0.000*	0.83	0.000*	0.57	0.001*
MRI and Height	0.44	0.002*	0.62	0.017*	-0.02	0.89
MRI and BSA	0.73	0.000*	0.79	0.000*	0.41	0.02
Japan SLV and Weight	0.96	0.000*	0.99	0.000*	0.85	0.000*
Japan SLV and Height	0.84	0.000*	0.99	0.000*	0.68	0.000
Japan SLV and BSA	1.00	0.000*	1.00	0.000*	1.00	0.000*
European SLV and Weight	0.96	0.000*	0.99	0.000*	0.85	0.000*
European SLV and Height	0.84	0.000*	0.94	0.000*	0.68	0.000
European SLV and BSA	1.00	0.000*	1.00	0.000*	1.00	0.000*

BSA: Body surface area. SLV: Standard liver volume. MRI: Magnetic resonance imaging. r: Pearson coefficient. (p<0.05*)

## Discussion

Hepatic volume has traditionally been measured by CT imaging, through the manual marking of hepatic contours and sum of the liver area in each axial section by a Radiologist. However, such manual methods are heavily operator dependent and require a considerable amount of time and attention. Automated and semi-automated forms of volumetric measurements were developed with various techniques and algorithms described [12]. The use of hepatic volume is a reliable and safe form when major resections are mandatory. The rational way to calculate the hepatic volume is to have the hepatic volume measured by the surgeon himself [13,14].

The results of different techniques have an excellent agreement, however, automated techniques can save around 30 minutes per patient.[7] Non-radiologists successfully performed precise volumetric measurements using these techniques [15]. The study of liver volume can be accurately calculated from CT or MRI, but the use of existing professional imaging software is often limited by costs, access, lack of trained radiology personnel, and specific hardware requirements. The standard Digital Imaging and Medical Communication (DICOM) allows the acquisition of volumetry by a technological support (tool coupled to the magnetic resonance software) or personal computer connected to the remote network of the Radiology hardware (CT or MRI scanner).

The volumetric analysis with CT uses three-dimensional images to analyse the volume of hepatic congestion in donors and recipients after preserving or not preserving the median suprahepatic vein revealing the reliability of this method of measurement and this is considered a gold standard method because the volumetric calculation is extremely accurate, providing great safety for the clinical and surgical follow-up of the patients [14,16].

Hepatic volume values measured by MRI and Japanese SLV formula revealed significant differences showing that this is not a very sensitive formula for the individuals of our study population. The comparison of the hepatic volume obtained by MRI with the SLV formula for European populations, showed no significant differences between the methods, being a more sensitive formula than the Japanese SLV.

There is a good similarity in the hepatic volume between the European, Japanese and Eastern Amazon population and, in the absence of MRI or CT imaging, it is possible to use SLV formulas as an approximated formula to measure the hepatic volume of our population.

Hepatic volume measurement precision is vital for surgical planning, operative viability, inoperable resections, and postoperative follow-up, avoiding postoperative complications such as the “Small for Size” syndrome, which is nothing more than a complication related to the hepatic graft weight and the weight of the receptor [17]. The study of the hepatic anatomy prior to liver transplantation is indispensable in surgical planning with a direct implication in liver transplantation results, making the pre-transplant liver volume study a routine procedure [13,18,19]. The methods used for volumetric liver study include USG, CT, and MRI. The MRI shows the bile ducts more clearly than the CT, ensuring a better planning and virtualization of the volume [20].

The MRI method, in the present study, showed a strong correlation when compared to both the European SLV formula and the Japanese SLV formula in males (Fig 5), with great similarities values found (r = 0.79 and p = 0, 0007). It is also noted that an increase in the mean values of hepatic volumetry measured by MRI (1648 ± 337.6) is accompanied by an increase in the mean values obtained by the SLV formula in the Japanese population (1405.1 ± 160.3).

In female individuals, the hepatic volume values assessed by MRI had a weak correlation with those obtained from Europe and Japanese SLV formulas. A previous study showed gender differences in liver measures, that was explained for smaller fat free mass found in women, even if they have same body weight comparing with male individuals [21]. In a study with healthy Chinese adults the estimated values of liver weight and body weight shows a positive correlation, gender dependent. The liver of males was heavier than females with same body weight [22].

Both SLV formulas use BSA data to estimate Liver volume, being the body weight a component of BSA calculation. When correlating the methods of measurement of hepatic volume with the anthropometric variables in the present study, the weight variable presented a strong to very strong correlation for all methods, regardless of gender, being the variable that presented better correlation with the MRI method and the applied SLV formulas. For females, the results of the SLV formula for Western Europe had a good correlation with weight, in contrast to the gold standard method.

The methods used to calculate hepatic volume have similarities, however the correlation between the data presented by MRI and the SLV formulas are gender dependent. In addition, the correlation between hepatic volume values of the individuals of both sexes measured by the SLV formulas for the Japanese and Western European populations indicated a close proximity between the measures.

## Conclusions

The results of the present study allow us to conclude that the use of MRI is a possible method for the analysis of hepatic volume, monitoring and evaluation of hepatic and hepatobiliary digestive surgeries, without causing any side effects to the patient, since the MRI is an innocuous examination. The results also demonstrate that the SLV formulas are not adequate for the studied population, and in a sex-stratified analysis, there is a clear need of adapting SLV equations for the Eastern Amazon population as the correlation had a better significance for men than for women.

We also noticed that there is a strong correlation between the weight and the BSA of the male individuals analysed with the hepatic volume measurements by MRI and the validated SLV formulas. In females, the correlation of hepatic measures obtained by SLV formulas are not observed in MRI scans. It shows the need of adaptations in formulas to apply in the Eastern Amazon population, taking in account the gender of the individuals to provide a safe applicable measurement of hepatic volume when imaging techniques are not available.

Finally, this research was conducted in only one region of Brazil, other studies like this should be conducted with the aim of verify the applicability of SLV formulas for Brazilian populations of other regions. If similar results were finding, it must reveal the need of adaptations of this validated SLV formulas to assure the patients safety.

## Supporting information

S1 File Volumetry study of Brazilian eastern amazon population data. (XLSX)
